# Association Between Life's Essential 8 Scores and Heart Failure: Insights From NHANES (2007–2020)

**DOI:** 10.31083/RCM39464

**Published:** 2025-09-24

**Authors:** Houde Duo, Shifeng Zhao, Yuhang Zhao, Alimujiang Awakeri, Hongchang Guo

**Affiliations:** ^1^The Sixth Clinical School of Medicine, Capital Medical University, 100013 Beijing, China; ^2^Department of Cardiovascular Surgery, Beijing Anzhen Hospital, Capital Medical University, 100013 Beijing, China

**Keywords:** heart failure, Life's Essential 8, chronic disease prevention, cardiovascular health, American Heart Association

## Abstract

**Background:**

Heart failure (HF) is a major public health concern due to the associated high morbidity, mortality, and growing economic burden. Thus, early prevention and identification of modifiable risk factors are crucial to reducing the prevalence of HF. The American Heart Association has recently introduced the Life's Essential 8 (LE8) metrics, which offer a comprehensive framework for assessing cardiovascular health. This research aims to investigate the association between the LE8 metrics and the prevalence of HF among the American population, exploring whether the LE8 metrics are associated with HF prevalence and can contribute to the risk stratification of HF in public health settings.

**Method:**

The present cross-sectional study utilized data from the National Health and Nutrition Examination Survey (NHANES) 2007–2020, including participants aged ≥20 years with complete data on HF and the LE8 metrics. Multiple logistic regression analysis was conducted to evaluate the relationship between LE8 and the prevalence of HF. Subgroup analyses combined with interaction tests were utilized to investigate potentially affecting factors. Furthermore, the dose-response association was analyzed using smooth curve fitting, while the receiver operating characteristic (ROC) curve was employed to examine the predictive performance of the LE8 metrics in HF patients.

**Results:**

A dose-response reverse linear association was identified between the LE8 scores and HF prevalence within the American population. When comparing the group with the lowest LE8 score to that with the highest score, the latter exhibited a 70% decrease regarding HF prevalence (odds ratio (OR) = 0.30; 95% confidence interval (CI), 0.22–0.43; *p* < 0.01) in the fully adjusted model. Moreover, variables including physical activity, nicotine exposure, sleep health, body mass index (BMI), and plasma glucose were identified as independently associated with the prevalence of HF.

**Conclusion:**

Higher LE8 scores were associated with a lower prevalence of HF, suggesting that the LE8 metrics may be a useful tool for identifying at-risk individuals in population health.

## 1. Introduction 

Heart failure (HF) denotes a multifactorial clinical syndrome stemming from irregularities in cardiac structure or functional performance [1]. It is typically characterised by dyspnoea and decreased exercise tolerance [2], significantly impacting patients’ quality of life. While a decline has been observed in the age-standardized incidence of HF, the total incidence of HF is increasing due to aging populations [1, 3]. This trend is similar to that observed in developing countries [4]. HF also places a substantial burden on public health [5], with projected healthcare financial outlays for HF within the United States exceeding $70 billion by 2030 [6]. These statistics highlight the crucial importance of HF prevention.

Life’s Simple 7 (LS7), a project launched by the American Heart Association (AHA) in 2010, aims to enhance cardiovascular health (CVH) among the general population [7]. LS7 comprises seven metrics (diet, physical activity, smoking, body mass index (BMI), fasting blood glucose, total cholesterol, and blood pressure), categorised as poor, intermediate, or ideal [7]. Ideal CVH is achieved when all metrics are at ideal levels. Multiple studies have indicated that a high LS7 score correlates with reduced HF incidence [8, 9, 10]. However, LS7 exhibits certain limitations. The prevalence of an ideal CVH within the American populace is below 1% [11]. Moreover, the scoring criteria are less sensitive to both interindividual variability and intraindividual changes [12]. To address the constraints of LS7, AHA introduced an updated framework for assessing ideal CVH in 2022, termed Life’s Essential 8 (LE8). LE8 incorporates sleep as an additional metric and enhances the scoring algorithm for existing metrics [12]. To our knowledge, no research has investigated the association between LE8 and HF within the American population. This cross-sectional study, based on a U.S. population, utilised National Health and Nutrition Examination Survey (NHANES) data from 2007–2020 that included individuals aged 20 and above, aiming to estimate the connection linking LE8 to the prevalence of HF and further to assess the predictive ability of LE8 for HF.

## 2. Methods

### 2.1 Study Population

**Fig. 1.**
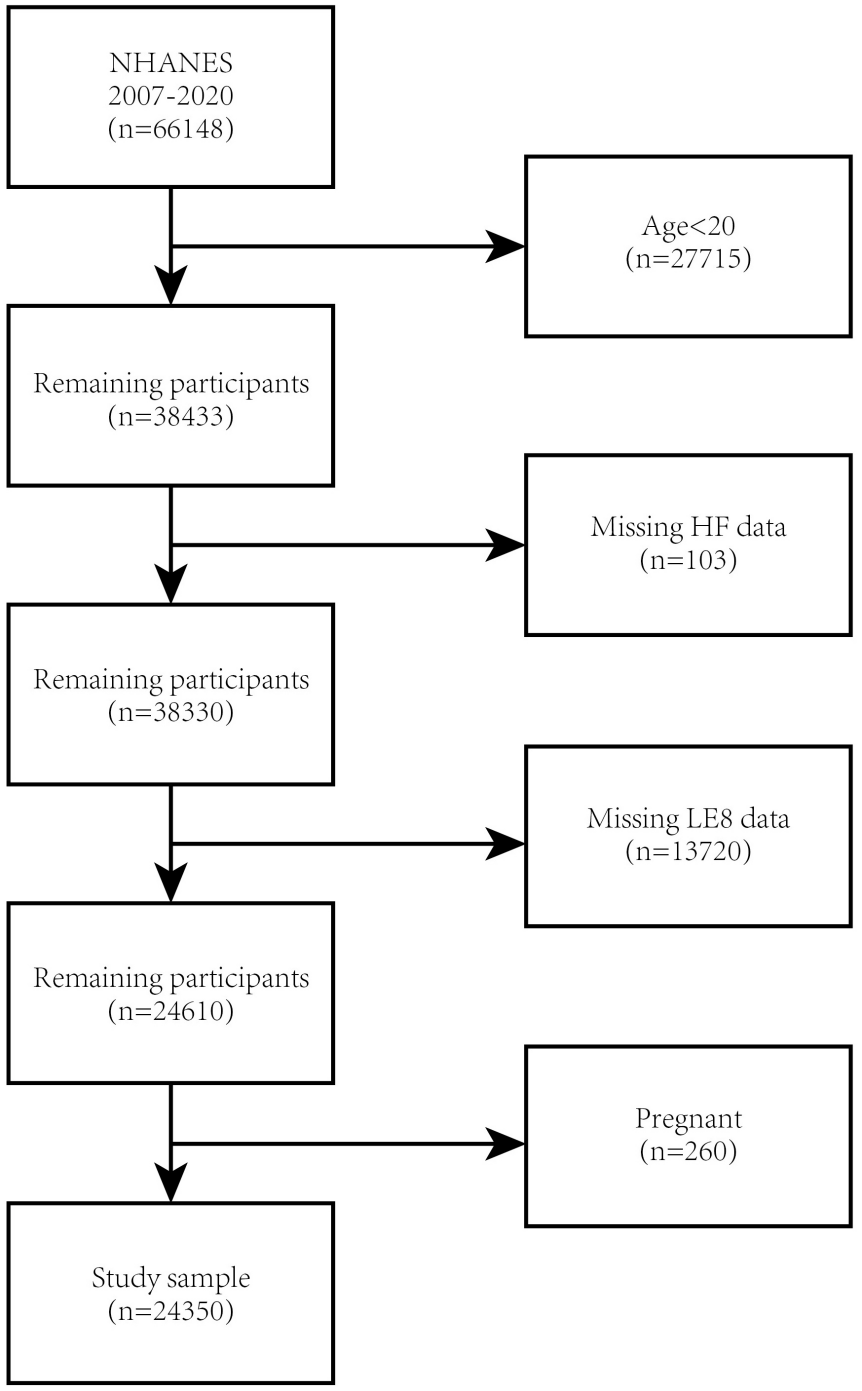
**Flowchart of participant selection**. NHANES, National Health and Nutrition Examination Survey; HF, heart failure; LE8, Life’s Essential 8.

NHANES is an ongoing cross-sectional survey to obtain nationally representative non-institutionalized samples [13]. Authorisation for the NHANES study was obtained from the Research Ethics Review Board of the NCHS, and written informed consent was acquired from all participants. This research utilised the NHANES dataset from 2007 to 2020. A total of 75,402 subjects participated throughout this continuous period. Exclusion criteria encompassed individuals who were under 20 years of age (n = 27,715), missing HF data (n = 103), missing LE8 data (n = 13,720), and pregnant (n = 260) (Fig. 1). The final cohort of our study comprised 24,350 eligible participants.

### 2.2 Outcome Variable: HF

HF data were collected using the variable MCQ160B in the MCQ questionnaire. Individuals who respond “yes” to the question “Have you ever been informed that you had congestive HF?” are considered to have HF. The lack of data on B-type natriuretic peptide, cardiac troponin, N-terminal proBNP, and echocardiography within the NHANES database justifies the utilisation of the questionnaire as a criterion for diagnosis for HF in NHANES participants. Previously published NHANES-based articles support this approach [14, 15, 16].

### 2.3 Exposure Variable: LE8

The LE8 score comprises four health behaviours (diet, physical activity, nicotine exposure, and sleep health) and four health factors (BMI, blood glucose, blood lipids, and blood pressure). The algorithm utilized for determining the LE8 scores based on the NHANES indicators is documented in **Supplementary Table 1**. A score of LE8 ≥80 is considered ideal, 50–79 is considered medium, and ≤49 is considered poor [12].

The Dietary Approaches to Stop Hypertension (DASH) Score was utilised to calculate dietary indicators [17]. The constituents and scoring criteria of the DASH Score can be found in **Supplementary Table 2** [17]. The DASH Score was estimated using data collected via two 24-hour dietary recalls collected through self-reported questionnaires (individuals with data from only one day are excluded). Data concerning the history of diabetes and medication, nicotine exposure, sleeping hours, as well as physical activity, were also gathered via self-reported questionnaires. Participants’ height, weight, and blood pressure are measured during the physical examination. BMI was obtained by dividing the weight in kilograms by the height squared, measured in meters (m^2^). The assessment of blood lipids, plasma glucose, and glycosylated hemoglobin type A1C (HbA1c) was conducted in central laboratories using collected blood samples.

### 2.4 Study Covariates

Covariates with the potential to impact the interplay involving LE8 and HF were taken into consideration, including gender (male, female), age (years), race (Mexican American, Non-Hispanic White, Non-Hispanic Black, and Other race), marital status (married/cohabitation, divorced/separated/widowed/unmarried), education level (less than high school, high school graduate, and beyond high school), and poverty ratio (<1.3, 1.3–3.49, and ≥3.5).

### 2.5 Statistical Analysis

NHANES sampling weights were used in all statistical analyses, factoring in the intricate multi-stage survey design. Baseline characteristics were split into two groups according to participants’ HF status. Continuous variables were summarised as mean (Mean ± SD), and categorical variables were described as percentages. A multivariate logistic regression analysis was employed to investigate the relationship between LE8 and HF, utilising three distinct models: Model 1 (unadjusted variables), Model 2 (adjusted for gender, age, and race), and Model 3 (adjusted for all covariates). The participants were stratified into subgroups according to age, gender, race, educational attainment, marital status, and poverty ratio for the purpose of conducting subgroup analyses, and interaction analyses were conducted accordingly. Smooth curve fitting was performed after adjusting for covariates to evaluate the dose-response relationship. The predictive utility of LE8 for HF was evaluated through the generation of Receiver Operating Characteristic (ROC) curves. R (version 4.2.1, R Foundation for Statistical Computing, Vienna, Austria, https://www.R-project.org/) and EmpowerStats (version 4.0, X&Y solutions, Inc., Boston, Massachusetts, USA, https://www.empowerstats.net) were employed for all statistical computations, and *p*-values below 0.05 were deemed to indicate statistical significance.

## 3. Results

### 3.1 Baseline Characteristics

Our study involved 24,350 participants, adhering to the established inclusion and exclusion criteria. Table 1 presents the baseline characteristics of our volunteers, with HF as a stratified variable. The weighted mean age (SD) of the participants was 49.87 ± 17.39, including 12,426 females (weighted percentage 51.1%). The majority were non-Hispanic white (weighted percentage 67.3%). The weighted mean score (SD) of LE8 was 66.90 ± 14.10. There were 767 participants with HF (weighted percentage of 3.1%). Individuals with HF demonstrate distinct demographic characteristics when compared to those without this condition. This population is more frequently male, older, and predominantly non-Hispanic white. Additionally, they are more likely to be single or divorced and exhibit lower levels of educational attainment, as well as higher poverty ratios.

**Table 1.** **Baseline characteristics based on the presence of heart failure**.

		Overall (n = 24,350)	Non-HF (n = 23,583)	HF (n = 767)	*p*-value
Age		49.87 ± 17.39	49.33 ± 17.27	66.58 ± 11.97	<0.01
Gender					0.07
	Male	48.91	48.81	53.16	
	Female	51.09	51.19	46.84	
Race					<0.01
	Mexican American	8.49	8.57	4.78	
	Non-Hispanic White	67.29	67.17	72.69	
	Non-Hispanic Black	10.42	10.34	14.06	
	Other race	13.80	13.92	8.48	
Education					<0.01
	Less than high school	13.78	13.49	26.17	
	High school graduate	23.04	22.91	28.59	
	Beyond high school	63.18	63.60	45.24	
Poverty ratio					<0.01
	<1.3	21.45	21.19	32.53	
	1.3–3.49	33.26	33.01	43.95	
	≥3.5	45.29	45.80	23.51	
Marital status					<0.01
	Married or live with spouse	63.51	63.68	56.38	
	Single or separated	36.49	36.32	43.62	
Life’s Essential 8		66.90 ± 14.10	67.16 ± 14.04	58.65 ± 13.46	<0.01
Diet		42.80 ± 33.45	42.77 ± 33.45	43.83 ± 33.65	0.39
Physical activity		63.85 ± 45.38	64.53 ± 45.15	42.89 ± 47.51	<0.01
Nicotine exposure		71.23 ± 39.16	71.42 ± 39.18	65.46 ± 38.38	<0.01
Sleep health		81.71 ± 25.33	81.90 ± 25.18	75.81 ± 29.22	<0.01
Body mass index		60.61 ± 34.36	61.02 ± 34.25	48.27 ± 35.39	<0.01
Non-HDL		69.47 ± 30.63	69.23 ± 30.61	77.05 ± 30.30	<0.01
Blood glucose		74.97 ± 26.72	75.59 ± 26.48	55.93 ± 27.28	<0.01
Blood pressure		70.53 ± 31.60	70.87 ± 31.44	59.97 ± 34.37	<0.01

For continuous variables, data are reported as Mean ± SD, and the corresponding *p* values were calculated via a weighted linear regression model; for categorical variables, weighted percentages were used, with *p* values determined by the weighted chi-square test. HF, heart failure; Non-HDL, non-high-density lipoprotein Cholesterol.

### 3.2 Association Between LE8 and HF

This research reveals that, as LE8 scores increase, the prevalence of HF tends to decrease, indicating a negative association, as demonstrated in Table 2. Both Model 1 (unadjusted model) (OR = 0.96; 95% CI, 0.95–0.96, *p* < 0.01) and Model 2 (adjusted for gender, age, and race) (OR = 0.97; 95% CI, 0.96–0.97, *p* < 0.01) indicate a statistically significant association. In Model 3 (adjusting for all covariates), this significant association remains (OR = 0.97; 95% CI, 0.97–0.98, *p* < 0.01). This analysis reveals that for each one-unit increase in LE8, there is a corresponding 3% reduction in the risk of developing HF. The ideal LE8 score group was associated with a 70% reduction in the prevalence of HF relative to the poor LE8 score group (OR = 0.30, 95% CI, 0.22–0.43, *p* < 0.01).

**Table 2.** **Association of LE8 and heart failure**.

Exposure		Model 1 OR (95% CI)	*p*	Model 2 OR (95% CI)	*p*	Model 3 OR (95% CI)	*p*
LE8 continues		0.96 (0.95, 0.96)	<0.01	0.97 (0.96, 0.97)	<0.01	0.97 (0.97, 0.98)	<0.01
LE8 categories							
	Low	Ref.		Ref.		Ref.	
	Moderate	0.42 (0.35, 0.49)	<0.01	0.45 (0.38, 0.54)	<0.01	0.52 (0.44, 0.62)	<0.01
	High	0.12 (0.09, 0.17)	<0.01	0.22 (0.16, 0.31)	<0.01	0.30 (0.22, 0.43)	<0.01

Model 1: None covariates were adjusted; Model 2: Adjusted for age, gender and race; Model 3: Adjusted for age, gender, race, education level, poverty ratio, and marital status. LE8, Life’s Essential 8; OR, odd ratio; CI, confidence interval; Ref, reference.

### 3.3 Subgroup Analyses

**Fig. 2.**
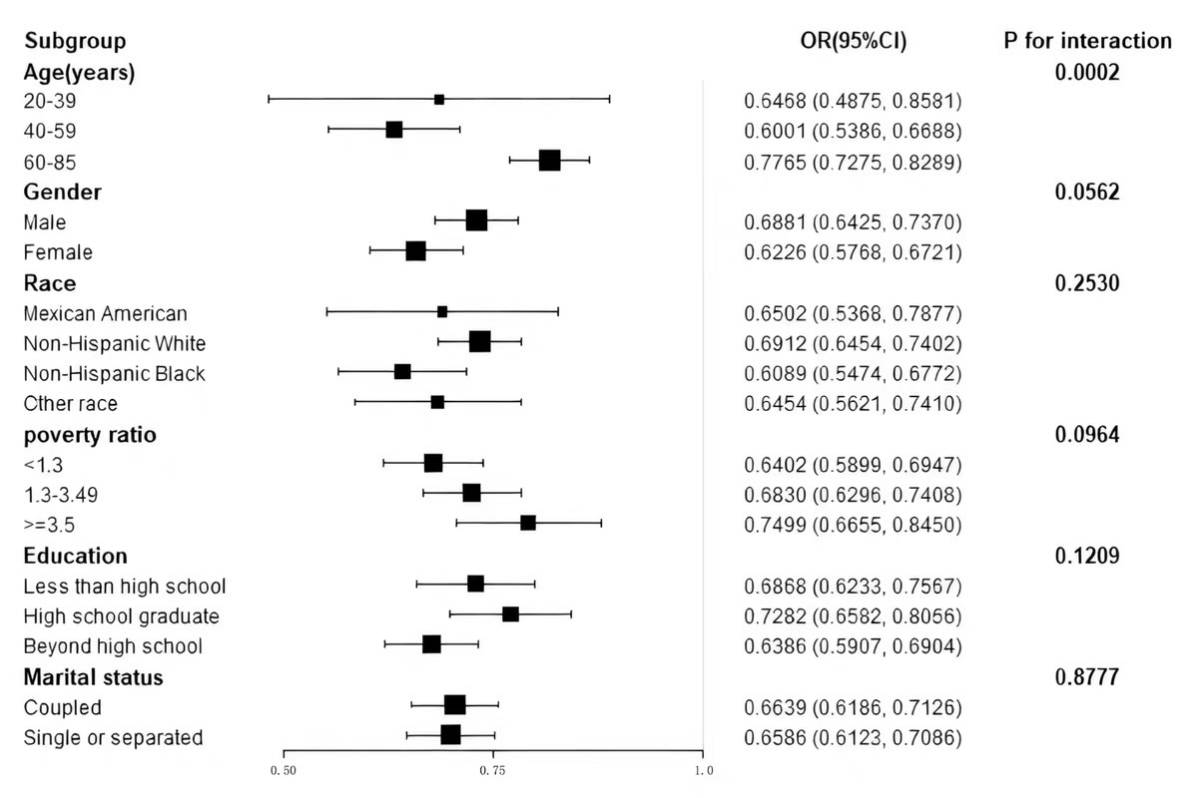
**Subgroup analysis of Life’s Essential 8 and heart failure association**. Association between LE8 scores and HF prevalence across subgroups of age, sex, race, education, marital status, and poverty ratio. Odds ratios were adjusted for relevant covariates.

To evaluate the consistency of the relationship across LE8 and HF prevalence throughout multiple demographic subgroups, we performed subgroup analyses considering various factors, including age, sex, race, education level, poverty ratio, and marital status. As shown in Fig. 2, there is an opposing trend observed in the values of LE8 scores and HF prevalence in every subgroup. The study results reveal a meaningful interaction pattern associated with LE8 scores and age (*p* for interaction = 0.0002). The association between LE8 and HF prevalence was significantly greater in older participants (aged 60–85 years) (OR per 10 scores, 0.7765; 95% CI, 0.7275–0.8289). Sex, race, education level, poverty ratio, and marital status did not significantly influence this relationship (*p* > 0.05 for all interactions). These findings indicate that the linkage relating LE8 scores to HF prevalence remains stable and robust across the various subgroups analysed.

### 3.4 Association of Individual LE8 Components and Risk of Incident HF

The connection between each LE8 component and HF prevalence was examined and detailed in Table 3. Upon adjustment for potential covariates, it was determined that ideal physical activity, nicotine exposure, ideal or medium sleep health, BMI, and plasma glucose were independently associated with HF prevalence.

**Table 3.** **Association of LE8 Components and heart failure**.

LE8 component		OR (95% CI)	*p*-value
Diet categorical			
	<50	Ref.	
	≥50, <80	0.97 (0.74, 1.27)	0.83
	≥80	1.21 (0.93, 1.57)	0.17
Physical activity categorical			
	<50	Ref.	
	≥50, <80	0.47 (0.21, 1.06)	0.07
	≥80	0.64 (0.50, 0.82)	<0.01
Nicotine exposure categorical			
	<50	Ref.	
	≥50, <80	0.92 (0.69, 1.23)	0.59
	≥80	0.64 (0.47, 0.87)	<0.01
Sleep health categorical			
	<50	Ref.	
	≥50, <80	0.63 (0.45, 0.89)	<0.01
	≥80	0.55 (0.40, 0.75)	<0.01
Body mass index categorical			
	<50	Ref.	
	≥50, <80	0.46 (0.36, 0.57)	<0.01
	≥80	0.40 (0.28, 0.56)	<0.01
Non-HDL categorical			
	<50	Ref.	
	≥50, <80	0.85 (0.60, 1.19)	0.34
	≥80	1.14 (0.94, 1.38)	0.18
Blood glucose categorical			
	<50	Ref.	
	≥50, <80	0.37 (0.28, 0.49)	<0.01
	≥80	0.22 (0.17, 0.30)	<0.01
Blood pressure categorical			
	<50	Ref.	
	≥50, <80	0.93 (0.77, 1.12)	0.43
	≥80	0.80 (0.54, 1.01)	0.06

Age, gender, race, education level, poverty ratio, and marital status were adjusted. LE8, Life’s Essential 8; Non-HDL, non-high-density lipoprotein Cholesterol.

### 3.5 Smooth Curve Fitting and ROC Curves

**Fig. 3.**
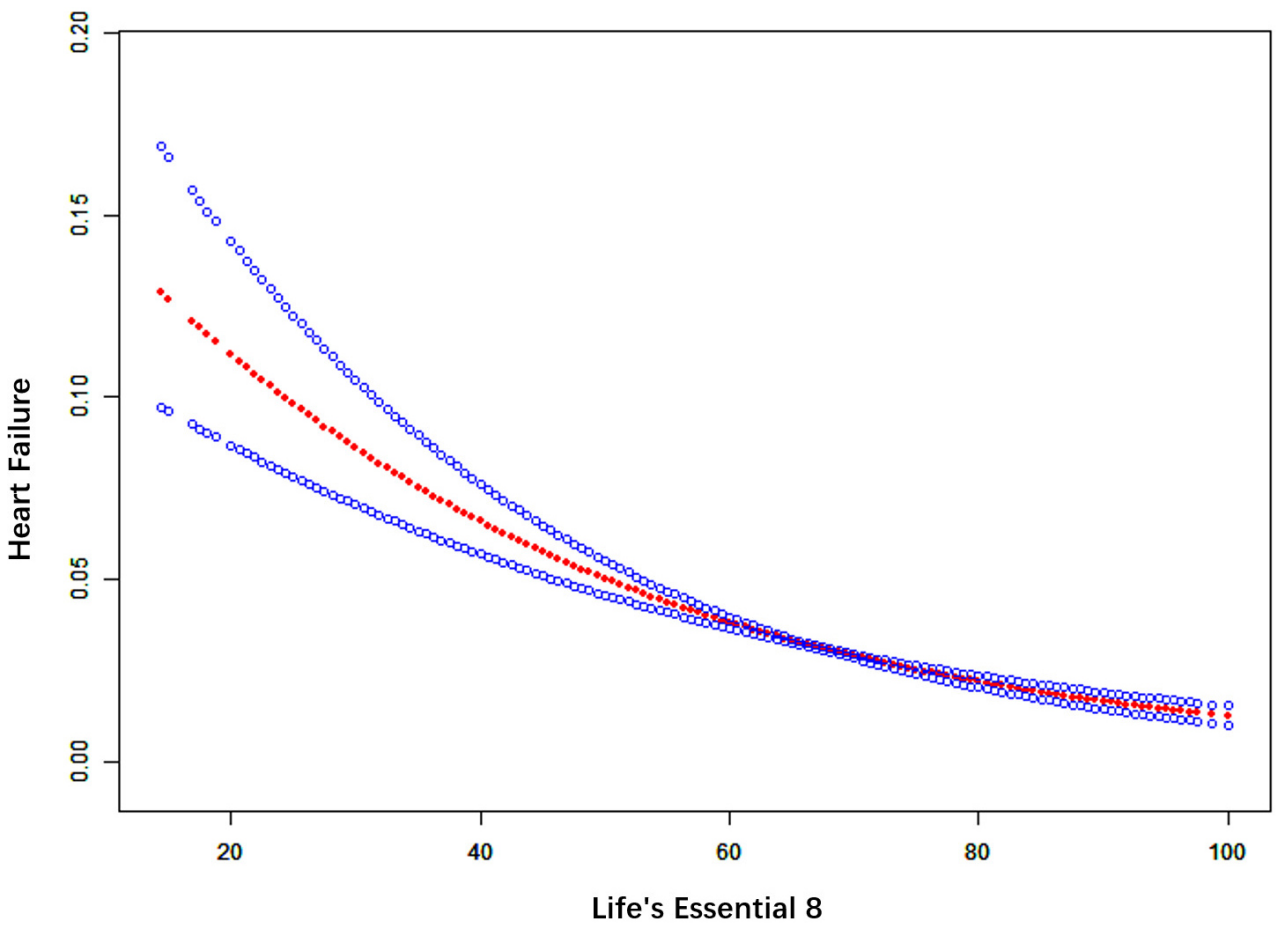
**Smooth curve fitting for LE8 and HF**. The red bands represent the smooth curve fit between variables. Blue bands represent the 95% confidence interval from the fit.

**Fig. 4.**
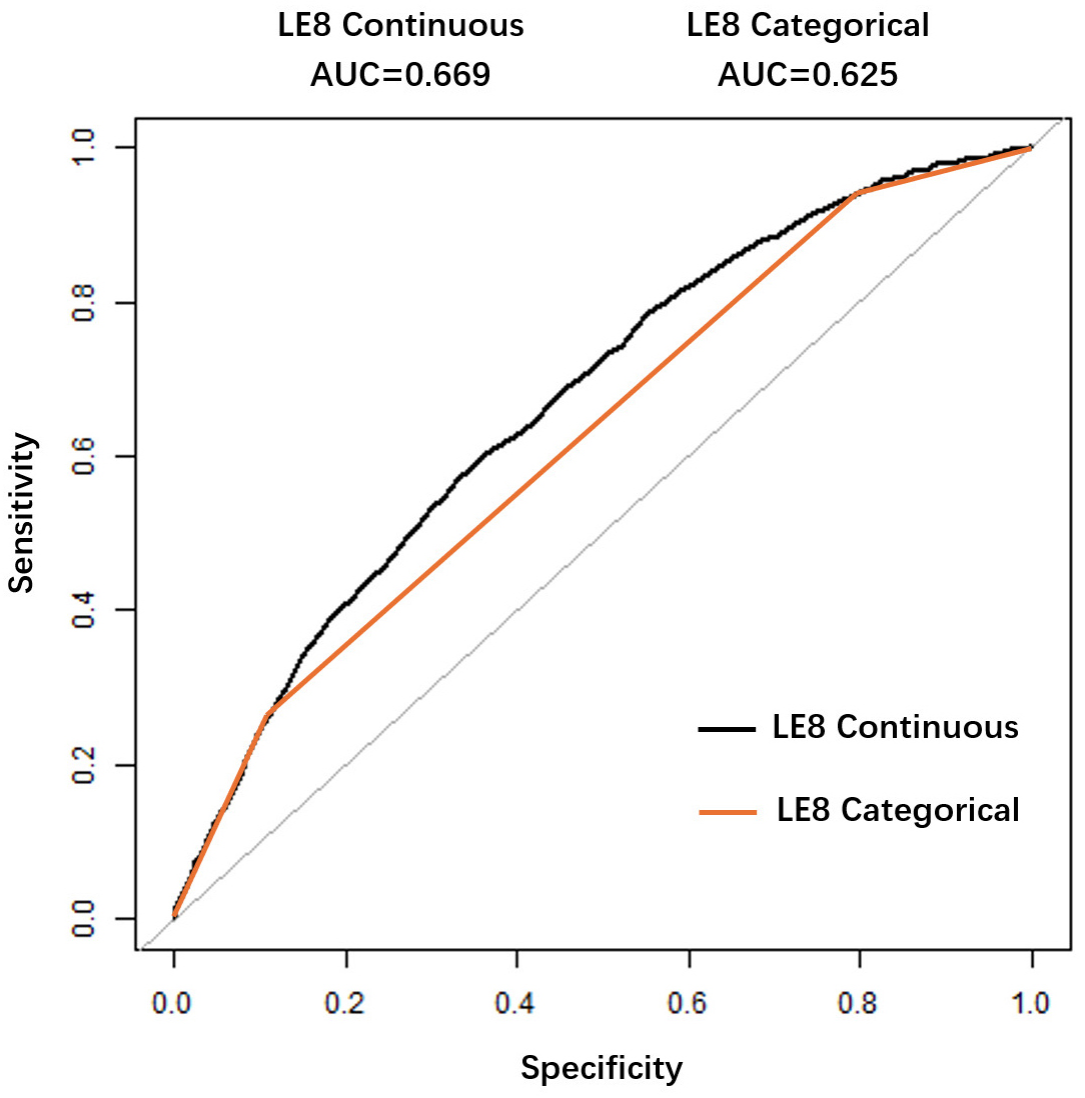
**ROC curve for evaluating the predictive value of Life’s Essential 8 for heart failure**. Receiver Operating Characteristic (ROC) curve for LE8 in predicting HF. The area under the curve (AUC) was 0.669. The optimal cutoff score was 62.19, with 60.10% sensitivity and 64.01% specificity.

A smooth curve fitting analysis was performed to further evaluate the association between LE8 and the prevalence of HF. Our findings revealed a linear dose-response relationship, as shown in Fig. 3, indicating that the risk of HF prevalence increases as LE8 scores decrease (*p* for non-linearity < 0.05). We also performed a ROC curve analysis to evaluate the forecasting efficacy of LE8 for HF. The area under the curve (AUC) serves as a quantitative measure of predictive accuracy, representing the overall effectiveness of the model in distinguishing among different classes. A higher AUC value indicates superior predictive performance, suggesting that the model is more adept at correctly identifying true positive and true negative instances. Conversely, a lower AUC value suggests reduced predictive capability. In this study, we determined the optimal threshold value to be 62.19—a critical juncture that delineates the trade-offs between sensitivity and specificity inherent in the predictive model. This threshold indicates that LE8 scores below this value still retain a degree of predictive significance for HF, as evidenced by the corresponding sensitivity and specificity metrics of 60.10% and 64.01%, respectively. These results underscore the complex link between LE8 scores and their predictive utility. A comprehensive presentation of the results, including both continuous and categorical representations of LE8, is provided in Fig. 4, which facilitates a deeper understanding of the model’s performance across different data structures.

## 4. Discussion

In this large-scale observational analysis with a sample of 24,350 participants, we investigated the relationship between LE8 and HF. The results revealed a negative association between LE8 scores and HF prevalence. Following adjustments for potential covariates, the subgroup analyses and interaction effect analyses revealed a consistent and robust trend in the association across diverse populations, underscoring the reliability of the observed relationship. Notably, the effect was more pronounced among the elderly, indicating that age might significantly impact the modulation of this association. Among the various components of LE8, factors such as physical activity, nicotine exposure, sleep health, BMI, and plasma glucose levels emerged as particularly significant contributors to the prevention of HF. These findings highlight the multifaceted nature of HF prevention and suggest that targeted interventions addressing these risk factors could yield substantial benefits, especially for older adults.

This study provides the first U.S. population-level evidence linking LE8—the AHA’s updated cardiovascular health metric incorporating sleep—to reduced HF prevalence. Key novel insights include: (1) identification of sleep health as independently protective against HF even at moderate levels, (2) validation of LE8’s superior clinical utility over prior LS7 metrics for HF risk stratification, and (3) delineation of modifiable LE8 components (physical activity, nicotine, BMI, glucose) as priority targets for prevention. Previous studies have reported the association of LS7 with HF through various epidemiological methodologies and across diverse target populations [8, 9, 10]. However, the components of LS7 were categorised as poor, moderate, and ideal [7], which inadequately addresses the evaluation of dose-response relationships. The relationship between sleep health and HF has been validated in multiple studies [18, 19, 20], yet LS7 did not incorporate this factor. Consequently, it is necessary to assess the relationship between newly introduced LE8 and HF prevalence. Recent studies have indicated a correlation between higher LE8 scores and improved CVH. For example, a cross-sectional study conducted by Sun *et al*. [21] within the American population (n = 19,951) demonstrated that elevated LE8 scores were correlated with decreased all-cause and cardiovascular mortality. Comparable findings were observed by Zhang *et al*. [22] in a prospective cohort study conducted within the UK population. A prospective cohort study conducted by Isiozor *et al*. [23] involving Finnish middle-aged men (n = 1899) revealed a reverse correlation between LE8 scores and the risk of atherosclerotic cardiovascular disease and venous thromboembolism. While there has been some research examining the connection of LE8 with HF risk within Asian populations [24] and the UK population [25], there remains a lack of relevant studies focusing on the American population.

The findings of our study align with those of prior studies [8, 9, 10, 24, 25]. A healthier lifestyle is linked to a decreased prevalence of HF despite variations in how poor, moderate, and ideal health lifestyles are defined across these studies. Our research indicates a linear association connecting LE8 scores to the risk of HF prevalence, which is consistent across different genders, age groups, and ethnicities. This suggests that individuals from various populations could benefit from improving their LE8 scores.

To further guide the prevention of HF, we analysed the interaction of each component of LE8 and the prevalence of HF. After adjusting for age, gender, race, marital status, education, and poverty ratio, we found that physical activity, nicotine exposure, sleep health, BMI, and plasma glucose were independently associated with the prevalence of HF.

Previous studies have shown that engaging in physical activity reduces the likelihood of HF by mitigating chronic subclinical myocardial injury [26], reducing the incidence of subclinical abnormalities in left ventricular systolic and diastolic function [27] and lowering the left ventricular mass index [28], with a dose-response relationship [29, 30]. Our study indicates that only an ideal level of physical activity is linked to a reduced prevalence of HF. Nicotine exposure, including smoking [31, 32] and secondhand smoke exposure [33, 34, 35], is recognised as an autonomous risk factor contributing to HF. The results of our study indicate that only ideal levels of nicotine exposure are correlated with lower HF prevalence, consistent with previous studies [36, 37]. This highlights the essential role of complete smoking cessation as a preventive measure against HF. Moreover, these results underscore the importance of public health efforts aimed at reducing nicotine exposure. Sleep has been newly incorporated as a component in LE8, reflecting its growing recognition in CVH frameworks. While existing literature has increasingly associated sleep health as a standalone risk factor for HF [18, 19, 20], prior research was unable to identify a notable connection linking sleep health to the risk of HF incidence [24]. Our research provides new evidence, demonstrating that even moderate levels of sleep health contribute to a significant reduction in HF prevalence. These results underscore the necessity of addressing sleep health in both clinical and public health contexts, advocating for targeted interventions to optimise sleep as a feasible strategy for HF prevention. We identified BMI and plasma glucose levels as independent risk factors for the prevalence of HF, which is consistent with previous research [24]. Our research indicates that even minor improvements in sleep health, BMI, and plasma glucose levels—shifting from poor to moderate—can significantly decrease the risk of HF occurrence. Our study emphasises the potential of incremental lifestyle changes in these areas to bring about meaningful reductions in HF prevalence. Promoting better sleep hygiene, encouraging healthy weight management practices, and supporting blood sugar regulation are essential strategies for both public health and clinical interventions. These results stress the necessity for scalable, evidence-based initiatives aimed at fostering long-term improvements in these modifiable factors. Overall, our findings call for a holistic and evidence-driven approach to CVH, advocating for sustainable and scalable preventive strategies that address manageable exposure factors to mitigate the global burden of HF.

Numerous studies indicate that adhering to the Mediterranean diet helps reduce the risk of HF [38, 39, 40]. However, prior research that assessed dietary scores based on Mediterranean diet standards failed to find a significant link between a healthy diet and HF [24]. Therefore, despite the contradictory findings regarding the relevance of the Dietary Approaches to Stop Hypertension (DASH) diet and HF [41, 42], our research still utilised DASH scores for dietary score calculations. Unfortunately, we also did not identify a notable relationship linking diet scores and the prevalence of HF. Similarly, we were unable to identify a connection between non-high-density lipoprotein (non-HDL) and HF prevalence, which is consistent with previous research. High non-HDL levels have shown connections to higher frequencies of cardiovascular events [43, 44] and all-cause mortality [45]. The PCSK9-LDLR axis has been linked to adverse outcomes in HF patients [46]. However, the utilisation of Rosuvastatin in HF patients did not result in improved patient outcomes [47, 48]. Further investigation is required to examine the relationship involving non-HDL and HF. Blood pressure is a recognised independent risk factor for HF. Although we found some evidence suggesting that the prevalence of HF is lower in the group with ideal blood pressure compared to those with poor blood pressure, the distinction noted between the two groups did not reach statistical significance (*p* = 0.0637). This lack of significance may be attributable to the limitations inherent in cross-sectional studies. It may take time for hypertensive patients to develop HF; HF patients typically use medications to manage their blood pressure, which could impact our study’s ability to find a meaningful association between the two variables.

While our findings highlight LE8’s role in HF prevention, they do not directly advocate altered treatment for established HF. Instead, they reinforce integrating LE8 metrics (e.g., physical activity, sleep, nicotine cessation) into primary care for at-risk populations. For diagnosed HF patients, optimizing these factors remains aligned with guideline-directed management, though causality requires longitudinal validation. Thus, LE8 serves best as a preventive tool—targeting modifiable risks early may reduce incident HF, complementing but not replacing current therapies.

Our study possesses several strengths, including the implementation of a complex multistage probability sampling design and accounting for potential covariates, which enhance the reliability and representativeness of the results. Furthermore, the substantial sample size and subgroup analyses enhance the robustness of our findings across various populations. Nonetheless, certain restrictions must be acknowledged. Firstly, given that our study is cross-sectional, we can identify an association between LE8 scores and HF prevalence but cannot infer causation. Secondly, a significant portion of participants lacked dietary data, which reduced the sample size and introduced potential bias.

Another important limitation of our study concerns the use of self-reported HF diagnosis via a single questionnaire item from NHANES. Although this method is commonly adopted in epidemiological analyses and supported by precedent NHANES-based studies, it remains vulnerable to misclassification bias. Participants may underreport or fail to recall a diagnosis, especially in subclinical or undiagnosed cases. Such misclassification is likely non-differential, which may bias associations toward the null, potentially underestimating the strength of the true relationship between LE8 and HF. Future studies incorporating clinical biomarkers or echocardiographic confirmation could provide more robust validation.

## 5. Conclusion

This study identified an inverse association between LE8 scores and HF prevalence in a nationally representative U.S. population. While causality cannot be inferred due to the cross-sectional nature of NHANES data, LE8 may serve as a practical metric for HF risk assessment in population health surveillance. Prospective studies are warranted to validate these findings.

## Availability of Data and Materials

The data sets generated and analyzed during the current study are available in the NHANES Datasets, https://wwwn.cdc.gov/nchs/nhanes/Default.aspx.

## Supplementary Material

Supplementary material associated with this article can be found, in the online version, at https://doi.org/10.31083/RCM39464.
