# Supplementary material for: Association Between Life's Essential 8 Scores and Heart Failure: Insights From NHANES (2007–2020)
Source: Rev Cardiovasc Med. 2025 Sep 24;26(9):39464. doi: 10.31083/RCM39464 (PMC12516766; doi:10.31083/RCM39464)
Supplement: Supplementary file 1 [file 2153-8174-26-9-39464-s1.docx]

Supplemental Table 1. Definition and scoring method for Life’s Essential 8.

| **Domain** | **CVH Metric** | **Method of Measurement** | **Quantification of CVH Metric – Adults**  **(≥20 Years)** | **Quantification of CVH Metric –Children**  **(Up to 19 Years*)** |
| --- | --- | --- | --- | --- |
| **Health Behaviors** | **Diet** | **Measurement:** Self-reported daily intake of a DASH-style eating pattern  **Example tools for measurement:** DASH diet score (populations) | Quantiles of DASH-style diet adherence    Scoring (Population):  Points Quantile  100 ≥95th %ile (top/ideal diet)  80 75th – 94th %ile  50 50th – 74th %ile  25 25th – 49th %ile  0 1st – 24th %ile (bottom/least ideal quartile) | Quantiles of DASH-style diet adherence; ages 2-19    Scoring (Population):  Points Quantile  100 ≥95th %ile (top/ideal diet)  80 75th – 94th %ile  50 50th – 74th %ile  25 25th – 49th %ile  0 1st – 24th %ile (bottom/least ideal quartile) |
|  | **Physical activity** | **Measurement:** Self-reported  minutes of moderate or vigorous  physical activity per week    **Example tools for**  **Measurement:**  NHANES PAQ-K questionnaire | **Metric:** Minutes of moderate (or greater) intensity  activity per week    **Scoring:**  Points Minutes  100 ≥150  90 120 – 149  80 90 – 119  60 60 – 89  40 30 – 59  20 1 – 29  0 0 | **Metric:** Minutes of moderate (or greater) intensity  activity per week; ages 2-19 years    **Scoring:**  Points Minutes  100 ≥420  90 360 – 419  80 300 – 359  60 240 – 299  40 120 – 239  20 1 – 119  0 0 |
|  | **Nicotine exposure** | **Measurement:** Self-reported use  of cigarettes or inhaled nicotine  delivery system  **Example tools for** **measurement:**  NHANES SMQ | **Metric:** Combustible tobacco use and/or inhaled  NDS use; or secondhand smoke exposure    **Scoring:**  Points Status  100 Never smoker  75 Former smoker, quit ≥5 yrs  50 Former smoker, quit 1 - <5 yrs  25 Former smoker, quit <1 year,  or currently using inhaled NDS  0 Current smoker    Subtract 20 points (unless score is 0) for living with active indoor smoker in home | **Metric:** Combustible tobacco use and/or inhaled  NDS use, or secondhand smoke exposure; ages 12-19  **Scoring:**  Points Status  100 Never tried  50 Tried any nicotine product, but >30 days ago  25 Currently using inhaled NDS  0 Current combustible use (any within 30 days)    Subtract 20 points (unless score is 0) for living with active indoor smoker in home |
|  | **Sleep health** | **Measurement:** Self-reported  average hours of sleep per night    **Example tools for** **Measurement:** “On average, how many hours of sleep do you get per night?”  Consider objective sleep/actigraphy data from wearable technology, if available | **Metric:** Average hours of sleep per night    **Scoring:**  Points Level  100 7 – <9  90 9 - <10  70 6 - <7  40 5 - <6 or ≥10  20 4 - <5  0 <4 | **Metric:** Average hours of sleep per night; ages 16-19    **Scoring:**  Points Level  100 Age-appropriate optimal range  90 <1 hr above optimal range  70 <1 hr below optimal range  40 1 - <2 hrs below or ≥1 hr above optimal  20 2 - <3 hrs below optimal range  0 ≥3 hrs below optimal range |
| **Health Factors** | **Body mass index** | **Measurement:** Body weight (kg) divided by height squared (m2)    **Example tools for** **Measurement:** Objective measurement of height and weight | **Metric:** Body mass index (kg/m2)    **Scoring:**  Points Level  100 <25  70 25.0 – 29.9  30 30.0 – 34.9  15 35.0 – 39.9  0 ≥40.0 | **Metric:** BMI percentiles (%iles) for age and sex; ages 2-19    **Scoring:**  Points Level  100 5th -- <85th %ile  70 85th -- <95th %ile  30 95th %ile -- <120% of the 95th %ile  15 120% of the 95th %ile -- <140% of the 95th %ile  0 ≥140% of the 95th %ile |
|  | **Blood lipids** | **Measurement:** Plasma total and HDL-cholesterol with calculation of non-HDL-cholesterol    **Example tools for** **Measurement:** Fasting or non-fasting blood sample | **Metric:** Non-HDL-cholesterol (mg/dL)  **Scoring:**  Points Level  100 <130  60 130 – 159  40 160 – 189  20 190 – 219  0 ≥220    If drug-treated level, subtract 20 points | **Metric:** Non-HDL cholesterol (mg/dL); ages 6-19    **Scoring:**  Points Level  100 <100  60 100 - 119  40 120 - 144  20 145 - 189  0 ≥190    If drug-treated level, subtract 20 points |
|  | **Blood glucose** | **Measurement:** Fasting blood glucose or casual hemoglobin A1c    **Example tools for** **Measurement:** Fasting (FBG, HbA1c) or non fasting (HbA1c) blood sample | **Metric:** Fasting blood glucose (mg/dL) or  Hemoglobin A1c (%)    **Scoring:**  Points Level  100 No history of diabetes and FBG <100 (or HbA1c < 5.7)  60 No diabetes and FBG 100 – 125 (or HbA1c 5.7-6.4) (Pre-diabetes)  40 Diabetes with HbA1c <7.0  30 Diabetes with HbA1c 7.0 – 7.9  20 Diabetes with HbA1c 8.0 – 8.9  10 Diabetes with Hb A1c 9.0 – 9.9  0 Diabetes with HbA1c ≥10.0 | **Metric:** Fasting blood glucose (mg/dL) or  Hemoglobin A1c (%); ages 12-19    **Scoring:**  Points Level  100 No history of diabetes and FBG <100 (or HbA1c < 5.7)  60 No diabetes and FBG 100 – 125 (or HbA1c 5.7-6.4) (Pre-diabetes)  40 Diabetes with HbA1c <7.0  30 Diabetes with HbA1c 7.0 – 7.9  20 Diabetes with HbA1c 8.0 – 8.9  10 Diabetes with Hb A1c 9.0 – 9.9  0 Diabetes with HbA1c ≥10.0 |
|  | **Blood pressure** | **Measurement:** Appropriately measured systolic and diastolic blood pressure  **Example tools for** **Measurement:** Appropriately sized blood pressure cuff | **Metric:** Systolic and diastolic blood pressure (mm Hg)    **Scoring:**  Points Level  100 <120/<80 (Optimal)  75 120-129/<80 (Elevated)  50 130-139 or 80-89 (Stage I HTN)  25 140-159 or 90-99  0 ≥160 or ≥100  Subtract 20 points if treated level | **Metric:** Systolic and diastolic blood pressure (mm Hg) percentiles for ages 8-12 years. For ages ≥13 years, use adult scoring.    **Scoring:**  Points Level  100 Optimal (<90th %ile)  75 Elevated (≥90th -- <95th %ile or ≥120/80 mm Hg to <95th %ile, whichever is lower)  50 Stage 1 HTN (≥95th -- <95th %ile + 12mmHg, or 130/80 to 139/89 mm Hg, whichever is lower)  25 Stage 2 HTN (≥95th %ile + 12mmHg, or ≥140/90 mm Hg, whichever is lower)  0 SBP ≥160 or ≥95th %ile + 30mmHg systolic, whichever is lower; and/or DBP ≥100 or ≥95th %ile + 20 mm Hg diastolic  Subtract 20 points if treated level |

Supplemental Table 2. Nutrient Targets for DASH Score

| **Nutrient** | **DASH Diet Nutrient Compositionª** | **DASH Score Target** | **Intermediate Target** |
| --- | --- | --- | --- |
| Saturated fat | 6% of energy | 6% of energy | 11% of energy |
| Total fat | 27% of energy | 27% of energy | 32% of energy |
| Protein | 18% of energy | 18% of energy | 16.5% of energy |
| Cholesterol | 150 mg | 71.4 mg/1000 kcal | 107.1 mg/1000 kcal |
| Fiber | 31 g | 14.8 g/1000 kcal | 9.5 g/1000 kcal |
| Magnesium | 500 mg | 238 mg/1000 kcal | 158 mg/1000 kcal |
| Calcium | 1240 mg | 590 mg/1000 kcal | 402 mg/1000 kcal |
| Potassium | 4700 mg | 2238 mg/1000 kcal | 1534 mg/1000 kcal |
| Sodiumb | 2400 mg | 1143 mg/1000 kcal | 1286 mg/1000 kcal |

ª Based on a 2100-kcal diet. ᵇ Sodium targets based on recommendations from the Sixth Report of the Joint National Committee on Prevention, Detection, Evaluation, and Treatment of High Blood Pressure.
